# Two-Stage Segmentation Framework Based on Distance Transformation

**DOI:** 10.3390/s22010250

**Published:** 2021-12-30

**Authors:** Xiaoyang Huang, Zhi Lin, Yudi Jiao, Moon-Tong Chan, Shaohui Huang, Liansheng Wang

**Affiliations:** 1 Department of Computer Science, School of Informatics, Xiamen University, Xiamen 361005, China; xyhuang@xmu.edu.cn (X.H.); invicxmuls@stu.xmu.edu.cn (Z.L.); ydjiao@stu.xmu.edu.cn (Y.J.); lswang@xmu.edu.cn (L.W.); 2 School of Science and Technology, Hong Kong Metropolitan University, Homantin, Kowloon 999077, Hong Kong; tmtchan@hkmu.edu.hk

**Keywords:** distance transformation, two-stage, deep learning, medical image segmentation

## Abstract

With the rise of deep learning, using deep learning to segment lesions and assist in diagnosis has become an effective means to promote clinical medical analysis. However, the partial volume effect of organ tissues leads to unclear and blurred edges of ROI in medical images, making it challenging to achieve high-accuracy segmentation of lesions or organs. In this paper, we assume that the distance map obtained by performing distance transformation on the ROI edge can be used as a weight map to make the network pay more attention to the learning of the ROI edge region. To this end, we design a novel framework to flexibly embed the distance map into the two-stage network to improve left atrium MRI segmentation performance. Furthermore, a series of distance map generation methods are proposed and studied to reasonably explore how to express the weight of assisting network learning. We conduct thorough experiments to verify the effectiveness of the proposed segmentation framework, and experimental results demonstrate that our hypothesis is feasible.

## 1. Introduction

The atrium is a component of the heart, one of the most important organs of humans, and its operation is closely related to human health. Atrial fibrillation is a common and persistent arrhythmia. When it occurs, the body’s heartbeat will be fast and irregular, and the atria will not contract normally, which may cause thrombosis to block blood vessels and increase the risk of stroke and heart failure.

In order to confirm the location of the lesion or compare the structure of organs and tissues, medical image analysis usually requires a professional diagnostician to manually mark the target area to gain a deeper understanding of its anatomy. An important reason for the poor treatment of atrial fibrillation in existing studies is the lack of in-depth understanding of the anatomical structure of the atrium. Although the expert’s manual segmentation of medical images can reconstruct the atrium for further research, this requires experts to have professional knowledge and rich work experience, and the cost of training such a doctor is huge. Therefore, it is of great significance to use intelligent computer methods to automatically segment the atrial structure in medical images to assist doctors in researching and treating atrial fibrillation.

The traditional image research technology is mainly to manually design features and then use machine learning algorithms to classify image features. As an emerging branch of machine learning, deep learning transforms the original feature representation space into another space through feature transformation layer by layer, thereby making tasks such as recognition, classification, and segmentation easier [1,2,3,4]. Compared with traditional artificial methods to construct features, this way of learning samples through big data can better characterize the rich information inherent in the data. The success of deep learning in the computer vision domain has also brought many inspirations to medical image research. Many studies have made various attempts on medical image segmentation using deep learning and have achieved inspiring results [5,6,7,8,9].

However, a noticeable defect in medical imaging is that the partial volume effect of organs or tissues can easily lead to unclear edges or blurry edges that restrict precise segmentation [10]. Combining the significance of atrium segmentation, this paper explores how to use deep learning to strengthen the learning of features near the edge of ROI to improve the performance of the left atrium MRI segmentation.

In summary, our main contributions are as follows: Regarding the distance map as the learning weight of the edge region, we propose a new segmentation framework based on two-stage learning, specifically: (1) use a simple two-stage network as the basic network framework and design a branch in its first stage to incorporate distance map information; (2) we design and discuss three methods for generating distance maps with edges as the target to effectively express the weights used to guide deep learning; (3) in order to further optimize network learning, Distdice Loss is proposed to emphasize the contribution of distance map to network training. In the end, the Dice score and Assd of the method we constructed on the ASC data set are 94.10% and 0.82 mm, which are improved by 2.72% and 0.53 mm compared with the two-stage network, respectively; (4) moreover, experimental results on the dataset demonstrate that our network sets a new state-of-the-art performance on the left atrium MRI segmentation dataset.

## 2. Related Work

### 2.1. Two-Stage Learning

In addition to using the end-to-end one-stage training method to segment medical images, some scholars have made many attempts using the two-stage idea and achieved exciting results [11,12,13]. Two-stage learning usually implements rough segmentation in the first stage and then puts rough segmentation into the second stage to continue training. It allows the deep neural network to learn features more effectively and achieve precise segmentation by giving training instructions or using specific skills in the first or second stage. Tang et al. [14] used a fully convolutional neural network to roughly segment the liver area in the first stage and crop the CT sub-images into the input of the second stage. Based on this, an edge enhancement network is proposed to segment the liver and tumor at the same time more accurately. Boot et al. [15] proposed a novel deep learning method based on a two-stage target detector that combines the enhanced Faster R-CNN and Libra R-CNN structure for target detection. The segmentation network is placed on top of the previous structure to accurately extract and position various features (i.e., edges, shapes). Jiang et al. [16] proposed a two-stage cascaded U-Net, using a variant of U-Net as the first stage network to obtain a rough prediction. Then, in the second stage, increase the width of the network and use two decoders to improve performance. The prediction map is refined in the second stage by cascading the preliminary prediction map with the original input to take advantage of the automatic context. The results of these studies fully illustrate the potential of the two-stage in the field of image segmentation. We will follow the steps of these studies and use the advantages of the two-stage to improve the performance of segmentation.

### 2.2. Distance Transformation

The idea of distance transformation has been widely used in many fields, including computer vision [17], image analysis [18], pattern recognition [19], and so on. The distance transformation algorithm can be used for shape matching and interpolation, skeleton extraction, separation of glued objects, target refinement, etc. Distance transformation is generally used to transform binary images [20]. In the image space, the pixels in a binary image can be divided into background pixels and target pixels. Take the case where the target pixel is 1 as an example: the pixel value of the target area is equal to 1 and the pixel value of the background area is equal to 0. The distance image generated by the distance transformation is a grayscale image rather than a binary image. The gray value represented by each pixel in this gray image is the distance from that pixel to the nearest background pixel.

Suppose there is a binary image with a connected area, which is the target area. Let *P* stand for the target pixel set, *Q* stands for the background pixel set, and *D* stands for the distance map. Then the distance transformation can be defined as:

(1) D(x)=Min(distance(p, q), p⊆P, q⊆Q

**Figure 1:**
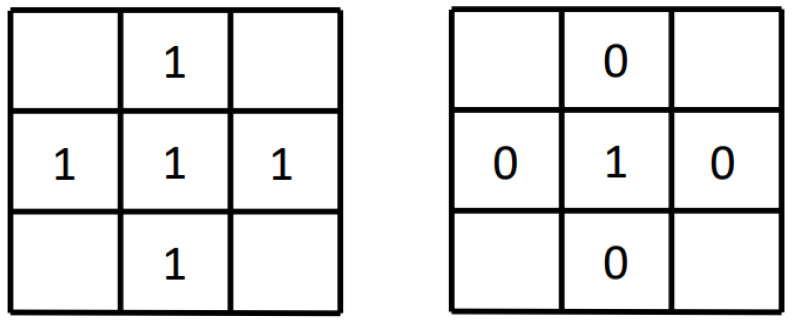
Internal points and isolated points.

First, the target pixels in the image are divided into external points, internal points, and isolated points. As shown in Figure 1, the left image is a schematic diagram of internal points and the right image is a schematic diagram of isolated points. Consider the center pixel and its four-neighborhood pixels: if the center pixel is the target pixel and its four-neighborhood pixels are also target pixels, it means that the center pixel is an interior point; if the center pixel is the target pixel and its four neighboring pixels are all background pixels, then this center pixel is an isolated point. Pixels that are neither internal points nor isolated points in the target area are boundary points.

Then calculate the internal points and non-internal points in the binary image to form point sets $C_{1}$ and $C_{2}$, respectively. For each internal point in $C_{1}$, the minimum distance from the pixel point in $C_{2}$ is calculated through the distance function and the set of these minimum distances constitutes $C_{3}$. Next, calculate the maximum value max and minimum value min in $C_{3}$. Taking a two-dimensional RGB image as an example, the gray value N obtained by conversion of each internal point can be expressed as:

(2) $$N=255*\;\left|C_{3}\left(p,\;q\right)-min\right|/\left|max-min\right|,\;p\subseteq P,\;q\subseteq Q$$

Here, $C_{3}\left(x,\;y\right)$ represents the shortest distance from the pixel in $C_{1}$ to the pixel in $C_{2}$. The distance function used in this paper is Euclidean distance and the distance transformation is Euclidean distance transformation. The formula for calculating the Euclidean distance is as follows:

(3) $$distance\left(p\left(x,\;y\right),\;q\left(x_{0},\;y_{0}\right)\right)=\sqrt{{\left(x-x_{0}\right)}^{2}+{\left(y-y_{0}\right)}^{2}},\;p\subseteq P,\;q\subseteq Q$$

## 3. Materials and Methods

### 3.1. Overall Network Architecture

**Figure 2:**
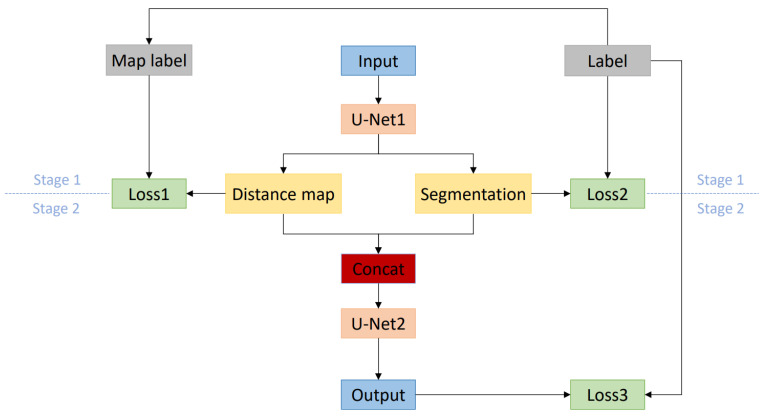
Schematic diagram of the overall network architecture.

In our method, the expected distance map after distance transformation with the edge of the left atrium as the target area is a grayscale image and also a weight map: the closer the area to the edge of the left atrium, the larger the pixel value is, and vice versa. It is conceivable that using such a weight map to participate in training can make the network pay more attention to the area near the edge of the left atrium. Moreover, the distance map is generated offline, not bringing additional overhead to training. Figure 2 is a schematic diagram of the overall architecture of the method. In the figure, “Label” represents the real label, “Map label” represents the distance map generated based on the real label, “Input” represents the training image, “Distance map” and “Segmentation”, respectively, represent the distance map and the rough segmentation result output by the network in the first stage, and “Output” represents the output of the second stage of the network.

The network training is divided into two stages. In the first stage, a variant of the U-Net [21] structure is used as the training network (U-Net1). It adds a branch parallel to the original U-Net up-sampling path. For clarity, the down-sampling path in the original U-Net is named image encoder and the up-sampling path is named image decoder. The newly added up-sampling branch is named distance decoder. The image encoder is composed of an initial convolutional layer and three basic modules. All convolutional layer kernels are equal in size to 3 and the number of channels in each layer is 16, 32, 64, 128 in sequence. The basic module of each layer consists of a convolution module and a down-sampling operation. Each convolution module is composed of two convolutional layers and the group normalization and ReLu activation function are inserted before each convolutional layer.

The decoding part of the network trained in the first stage has two branches: the image decoder and the distance decoder. These two decoders share the encoder from the image mentioned above. Before the feature map is down-sampled by the decoder, it will skip connection with the input of the encoder of the same level. The image decoder and the distance decoder are also composed of three basic modules, each of which is composed of a convolution module and an up-sampling operation. The up-sampling operation of the image decoder uses transposed convolution, while the up-sampling operation of the distance decoder uses trilinear interpolation. After connecting the final up-sampling results of the image decoder and the distance decoder in the channel dimension, they are input into the second-stage network (U-Net2) for training. The configuration of the second-stage network is the same as that of the first-stage network, but the distance decoder is deleted. Use the softmax function to output the final prediction.

### 3.2. Distance Map Generation

The primary purpose of this method is to obtain a learning weight map that can assist in the segmentation of the left atrium edge. When the pixel value of the target area in the label is 1 and the pixel value of the background area is 0, the distance map generated according to the label should satisfy that the closer the target edge area is, the larger the pixel value, and vice versa. Corresponding to the pixels in the original image, the pixel values in the distance map represent the strength that the network needs to learn. In order to find a distance map that can effectively represent the learning intensity, this section discusses three different ways of generating distance maps and then validates the performance of the three methods in subsequent experiments.

**Figure 3:**
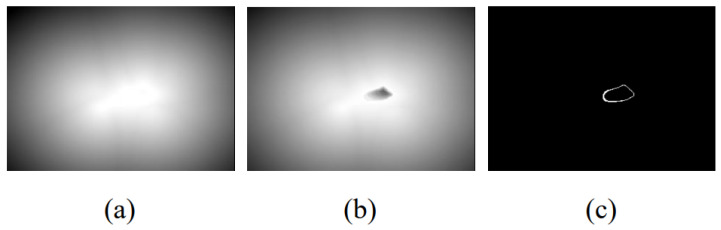
Schematic diagram of the distance map generated by the three methods. (**a**) Euclidean distance map; (**b**) Error compensation distance map; (**c**) Edge labeled distance map.

The processing step of the first method, named Method A, is to obtain the edge image of the left atrium and reverse it. Then, generate a distance map according to the Euclidean distance transformation. Next, the distance map is normalized to [0, 1] and the result of subtracting the distance map from 1 is used as the final distance map for supervision. At this time, the supervision distance map satisfies that the pixels close to the edge have a more enormous value. Figure 3a shows the distance map generated by this method.

The second method, called Method B, is derived from the paper [22]. First, we perform distance transformation on the real label area and then subtract the results from the maximum distance value generated. Take the absolute value of the above result and multiply it with the original label to generate an error compensation distance map. Second, reverse the original label and perform the same steps above to calculate the distance map inside the left atrium. Third, normalize the results generated in the first two steps separately and add them in voxel mode to obtain the final result. Figure 3b shows the distance map generated by this method.

In addition to the above two methods, we also explored Method C to generate distance maps. In the distance maps generated by Method A and Method B, the pixel value represents the distance to the target area. Assume an extreme situation: only the pixels in the target area are infinitely close to the target area and the other pixels are the opposite. Therefore, we tried a simple and extreme distance map: directly use the edge of the left atrium as a supervised label to guide the training of the distance decoder. Figure 3c shows the distance map generated by this method.

### 3.3. Loss Function

As shown in Figure 2, the proposed network framework needs to design three loss functions which are used for the first-stage image decoder branch training, the distance decoder branch training, and the second-stage training. The image decoder branch training is no different from regular segmentation, so the loss function is always set to Dice Loss [23]. We discussed two loss functions for the training of the distance decoder branch: Mean Absolute Error Loss (MAE Loss) and Mean Square Error Loss (MSE Loss) [24]. MAE Loss represents the sum of the absolute difference between the label and the prediction, while MSE Loss can represent the expectation of the square of the difference between the label and the prediction.

(4) $$\mathrm{MAE}\;\mathrm{Loss}=\frac{1}{N}\sum_{i=1}^{N}\left|y^{i}-p^{i}\right|$$

(5) $$\mathrm{MSE}\;\mathrm{Loss}=\frac{1}{N}\sum_{i=1}^{N}{\left(y^{i}-p^{i}\right)}^{2}$$

Compared with the general segmentation, the input of the second stage adds a distance map in addition to the original image. In order to emphasize the contribution of distance map to training, we propose Distdice Loss, which uses a distance map to give weight to each pixel based on Dice Loss:

(6) $$L_{Distdice}=-\frac{2\left|Y⋂P\right|\;*\;D}{\left|Y\right|\;+\;\left|P\right|}$$

In Formulas (4)–(6), *Y*, $y^{i}$ represent labels, *P*, $p^{i}$ represent the predictions output by the second-stage network, and *D* represents the distance map output by the first-stage distance decoder.

### 3.4. Dataset

The Atrial Segmentation Challenge (ASC) 2018 dataset is a public dataset for left atrium segmentation tasks. It used a total of 154 cases of 3D MRI data. The original resolution of the data is 0.625 × 0.625 × 0.625 mm³. The University of Utah (NIH/NIGMS Integrated Biomedical Computing Center (CIBC)) provided most of the data, and the rest came from multiple other institutes. All clinical data have been approved by institutional ethics. Each 3D MRI patient data are acquired using a clinical whole-body MRI scanner, and the patient data contain the original MRI scan and the corresponding left atrium annotation, which is manually marked by medical experts. The original MRI is grayscale, and the labels are in binary format. The data set is split into a training set and a test set, of which 100 patient data are used for training and 54 patient data are used for testing. Since the official test set is not available, our experiment re-adjusts the training set randomly to select 80 MRI scans for training and the remaining 20 MRI scans for evaluation.

### 3.5. Implementation Details

The experiment is based on Linux Ubuntu 16.04 LTS system and PyTorch deep learning framework. Each experiment uses a NVIDIA GeForce GTX 1080 Ti graphics card with 11G of memory. Before the experiment, the distance map was generated according to the three methods introduced in Section 3.2. The evaluation standard to measure the accuracy of prediction is the Dice similarity coefficient [25] and the average symmetric surface distance (Assd) [26].

All data are normalized, and a complete input image is randomly cropped according to the size of 232×232×32 and the batch size is set to 1. Gradient descent uses Adam optimizer, and the initial learning rate is $1\times 10^{-4}$. The update method of the learning rate is shown in Formula (7), where $\alpha_{0}$ is the initial learning rate and α is the current learning rate. In addition, *e* in Formula (7) is the current epoch and *N* is the maximum training epoch, which is set to 110. As the training progresses, the learning rate will slowly decay until it reaches zero.

(7) $$\alpha =\alpha_{0}\times {\left(1-\frac{e}{N}\right)}^{0.9}$$

## 4. Experimental Results

### 4.1. Effectiveness of Two-Stage Learning

In order to explore the performance of the one-stage network and the two-stage network, we compared the two networks based on experiments. The two-stage network is similar to the network structure shown in Figure 2, but the distance decoder branch is deleted. In other words, only the output of the first-stage image encoder is used as the second-stage input. The one-stage network is a classic 3D U-Net network, and its structure is the same as the second stage of the two-stage network.

**Figure 4:**
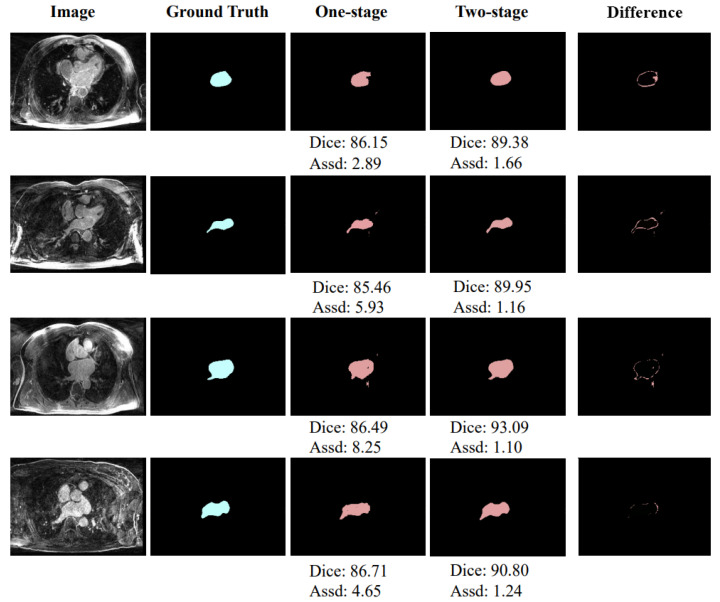
Exemplar segmentation results of the one-stage network and two-stage network.

As shown in Table 1, the two-stage achieves 5.04% and 8.12 mm improvements over the original 3D U-Net in the Dice score and Assd, respectively, which verifies the effectiveness of the two-stage learning. In addition, Figure 4 shows a schematic diagram of partial segmentation results and the segmentation difference between one-stage and two-stage methods. The units of Dice score and Assd in Figure 4 are % and mm, respectively. It can also be intuitively observed from the figure that the segmentation of the two-stage network is closer to the ground truth.

### 4.2. Effectiveness of Distance Map

The method designed in this paper is based on the idea that the distance map generated with the edge of the left atrium as the target has a more significant weight in the area close to the edge, which can guide the network to pay more attention to the edge and strengthen the learning of edge features. Therefore, finding a distance map that can reasonably represent the edge learning weight becomes a key point. This section compares and analyzes the three distance maps introduced in Figure 3 from an experimental point of view.

We compare the segmentation performance with different design choices and show the results in Table 2. The network structure used here has been described in detail in Section 3.1. From the table, we can observe that: (1) the three distance maps generated by Method A (Figure 3a), Method B (Figure 3b), and Method C (Figure 3c) bring 2.72%, 1.88%, and 1.98% improvements in average Dice score, and 0.53 mm, 0.43 mm, and 0.47 mm improvements in average Assd compared to the two-stage network, respectively; and (2) among the three methods, Method A has achieved the highest performance, which brings 0.84% and 0.74% improvements in average Dice score and 0.10 mm and 0.06 mm improvements in average Assd compared to Method B and Method C, respectively.

**Figure 5:**
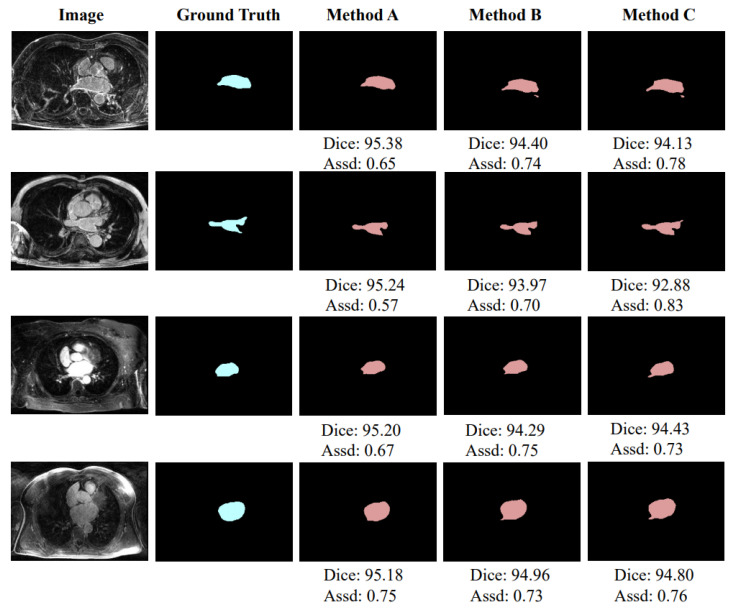
Exemplar segmentation results using different methods to generate distance maps.

As shown in Table 2, although Method C uses the edge of the left atrium as the distance map, it can also improve network performance, which can prove that the idea of using the distance map module is correct and feasible. However, the information provided by Method C is quite limited and cannot provide continuous information of strong and weak changes like a real distance map, so the performance of this method is not optimal. In addition, the performance of Method B is lower than that of Method A, and even slightly worse than that of Method C. The reason may be that, although the distance map generated by Method B can provide continuous information, the intensity of pixels at the same distance inside and outside the left atrium edge is asymmetrical, which may interfere with the learning of the network. The abovementioned results prove that the distance map generated by Method A can provide the most reasonable auxiliary information to help network learning. Figure 5 shows a schematic diagram of partial segmentation results. The units of Dice score and Assd in Figure 5 are % and mm, respectively.

### 4.3. Network Optimization

The method proposed in this paper is based on the network architecture shown in Figure 2, and the distance map generation method adopts Method A introduced in Section 3.2. Based on this, this section mainly explores the optimization process of this method.

**Figure 6:**
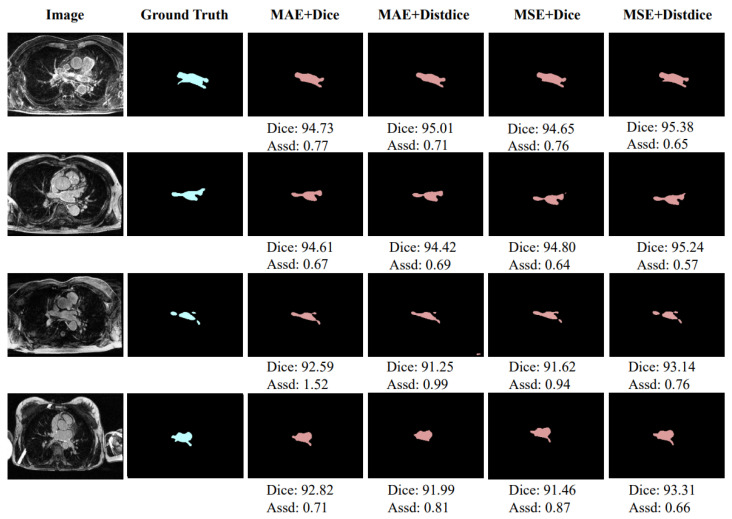
Exemplar segmentation results using different combination loss functions.

Table 3 shows the results of comparative experiments on using different loss function combinations to optimize training. The loss function used by the image decoder in the first stage is always Dice Loss. In Table 3, MAE Loss and MSE Loss represent the optional loss function used by the distance decoder in the first stage, and Dice Loss and Distdice Loss only represent the optional loss function for the second stage of network training. As shown in Table 3, when the distance decoder branch uses MSE Loss and the second-stage training of the network uses Distdice Loss, the highest segmentation level can be achieved, with an average Dice score of 94.10% and an average Assd of 0.82 mm. Figure 6 shows the segmentation of training using different combinations of loss functions. The units of Dice score and Assd in Figure 5 are % and mm, respectively.

### 4.4. Comparison of Other Methods

Tabel Table 4 summarizes the quantitative results of our proposed method and several state-of-the-art methods, including LG-ER-MT [27], DUWM [28], MC-Net [29], V-net [30], Bayesian V-net, and AJSQnet [31]. Among them, LG-ER-MT, DUWM, and MC-Net utilized the semi-supervised strategy with uncertainty prediction, while V-net, Bayesian V-net, and AJSQnet are trained by all labeled data, and Bayesian V-Net utilized the Bayesian network to adapt the vanilla V-Net. MC-Net has its best Dice of 90.34% and Assd of 1.77 mm in semi-supervised filed. For the other general methods, AJSQnet has the best Dice of 91.14% and V-Net of Bayesian version has the best Assd of 1.52 mm. However, it is worth noting that our proposed two-stage method guided by distance transformation further outperforms MC-Net, AJSQnet, and Bayesian V-Net in terms of both metrics Dice and Assd, and the corresponding scores are 94.10% and 0.82 mm. Our method brings 3.76%, 2.80%, and 2.96% improvements in average Dice score and 0.95 mm, 0.78 mm, and 0.70 mm improvements in average Assd compared to MC-Net, AJSQnet, and Bayesian V-Net, respectively.

## 5. Discussion and Conclusions

Medical images contain plentiful information, which is very suitable for using deep learning to mine valuable information. However, the crucial problem is that the edges of organ tissues should provide potential information as a boundary becomes visually blurred due to objective reasons such as the partial volume effect. Therefore, we aim to conduct meaningful experiments on the edge of medical images, and one idea worthy of expansion is distance transformation. In addition, two-stage learning has shown advantages in improving the network structure and facilitating training guidance, making it gradually become a research method of medical image segmentation that has attracted much attention.

Based on the above, we propose a two-stage segmentation method for medical images based on distance transformation. By using the edge of the left atrium as the target area for distance transformation, the obtained distance map can be used as a learning weight map to make the network pay more attention to the area near the edge of the organ. The training is divided into two stages in total. In the first stage, two branches are derived to predict the rough segmentation of the left atrium and the distance map, respectively, and the two are merged into the second stage of training to obtain accurate segmentation results. The experimental results proved that our idea is practical and effective.

There are still limitations in our study. On the one hand, our method has three loss functions: the first-stage image decoder training, the first-stage distance decoder training, and the second-stage training. This paper only discusses the loss functions of the first-stage distance decoder training and the second-stage training. In the future, we will focus on exploring the three loss functions for joint training and exploring the space of optimization models. On the other hand, this article only conducted experiments on left atrium MRI images. Other forms of medical images (such as X-ray, CT, etc.) are different from MRI images in terms of generation principles and image characteristics, which may affect the performance of the algorithm. The generalization ability of the algorithm in other organs and other forms of medical imaging needs further verification.

In conclusion, the method proposed in this paper takes advantage of the feature that the pixel value in the distance map obtained by the distance transformation will change with the distance from the target area. It improves the accuracy of image segmentation through a two-stage training method, which provides new ideas for exploring medical image segmentation.

## Figures and Tables

**Table 1:** Performance comparison between one-stage network and two-stage network.

Network	Dice (%)	Assd (mm)
One-stage (baseline)	86.34	9.47
Two-stage	91.38	1.35

**Table 2:** Performance comparison of distance map methods.

Network	Dice (%)	Assd (mm)
Two-stage	91.38	1.35
Design with Figure 3a	94.10	0.82
Design with Figure 3b	93.26	0.92
Design with Figure 3c	93.36	0.88

**Table 3:** Performance comparison experiment of loss function.

The First Stage		The Second Stage		Dice (%)	Assd (mm)
MAE Loss	MSE Loss	Dice Loss	Distdice Loss		
	✓	✓		93.08	1.01
✓			✓	93.12	1.00
✓		✓		93.57	0.95
	✓		✓	94.10	0.82

**Table 4:** Performance comparison of our method and compared methods.

Network	Dice (%)	Assd (mm)
LG-ER-MT [27]	89.62	2.06
DUWM [28]	89.65	2.03
MC-Net [29]	90.34	1.77
V-net [30]	90.25	1.91
Bayesian V-Net	91.14	1.52
AJSQnet [31]	91.30	1.60
Proposed	94.10	0.82

## Data Availability

Not applicable.
